# Supplementary material for: Tinnitus risk factors and its evolution over time
Source: Nat Commun. 2025 May 7;16:4244. doi: 10.1038/s41467-025-59445-3 (PMC12059016; doi:10.1038/s41467-025-59445-3)
Supplement: Supplementary file 1 — Supplementary Information [file 41467_2025_59445_MOESM1_ESM.pdf]

Tinnitus risk factors and its evolution over time
Supplementary materials

L.Hobeika[1,2,3,4], M.Fillingim[3,5], C.Tanguay-Sabourin[3,6,7], M.Roy[3,4,6], A.Londero[8], S.Samson[1,9,10], E.Vachon-Presseau[3,6,11]

1. Université Paris Cité, Institut Pasteur, AP-HP, Inserm, CNRS, Fondation Pour l'Audition, Institut de l'Audition, IHU reConnect, F-75012 Paris, France
2. Sorbonne Université, Institut du Cerveau - Paris Brain Institute - ICM, Inserm, CNRS, APHP, Hôpital de la Pitié Salpêtrière, Paris, France
3. Alan Edwards Centre for Research on Pain, McGill University, Montreal, Quebec, Canada
4. Department of Psychology, McGill University, Montreal, Quebec, Canada.
5. Integrated Program in Neuroscience, McGill University, Montreal, Quebec, Canada
6. Department of Anesthesia, Faculty of Medicine and Health Sciences, McGill University, Montreal, Quebec, Canada.
7. Faculty of Medicine, Université de Montréal, Montreal, Quebec, Canada.
8. Université Paris Cité, Institut Pasteur, AP-HP, Hôpital Lariboisière, Service ORL, INSERM, Fondation Pour l'Audition, IHU reConnect, F-75010 Paris, France
9. PSITEC – Psychologie : Interactions, Temps, Emotions, Cognition, Université de Lille, ULR 4072, Lille, France
10. Assistance Publique – Hôpitaux de Paris, GHU Pitié-Salpêtrière-Charles Foix, F-75013 Paris, France
11. Faculty of Dental Medicine and Oral Health Sciences, McGill University, Montreal, Quebec, Canada.

Figure S1: Features loadings (Pearson's *r* correlation coefficient between features and the models' scores) of the **A** Presence Risk Score model and **B** the Severity Risk Score model

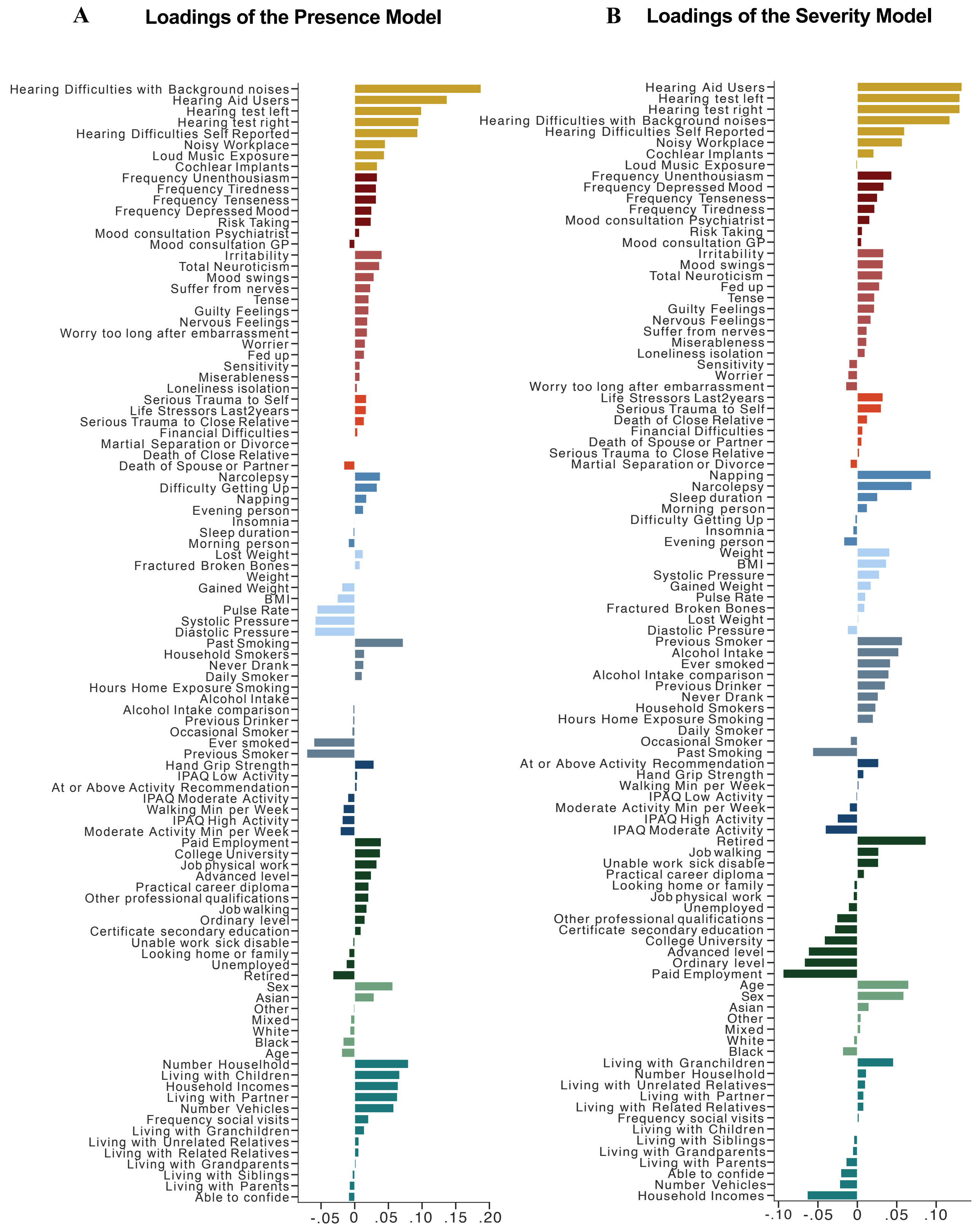

Figure S2: Sex stratified analysis. We tested the presence and severity models separately in women and men. Results did not evidenced differences in the cross-sectional or longitudinal analysis.

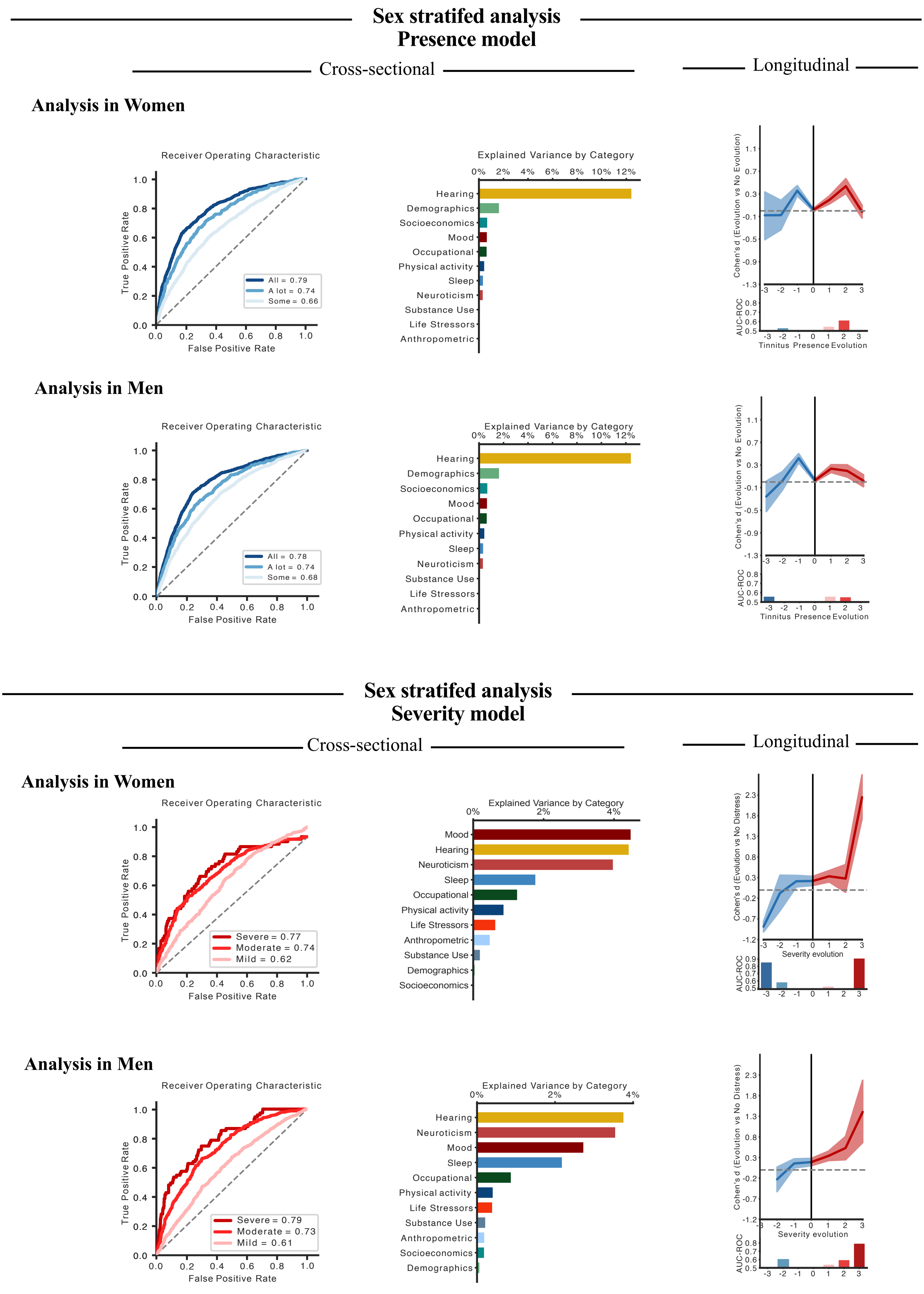

Figure S3: Validation of the presence and severity risk scores over ethnicities.

To ensure the validity of the presence and severity models on different ethnicities, we tested the performances of the models separately on groups of Asian, Black and White individuals. We tested participants which were not included in the rest of the analysis, using data of participants who did not had a full hearing evaluation at baseline visit but had one in the follow-up visit. We tested 498 Asian, 250 Black and 28,841 White individuals with the presence model, and 99 Asian, 41 Black and 6,934 White individuals with the severity model. The presence model **(A)** had good to excellent performances to classify individuals perceiving tinnitus a lot or all the time (except for the Asian "a lot of the time" group). The severity model **(B)** had good to excellent performances to classify individuals with moderate or severe distress (except for the Asian "moderate" group). Overall, the models had good performances for the extreme levels (all the time, or severe distress) for all ethnicities. The performances for the intermediate levels (A lot of the time, moderate distress) were good for Black and White individuals, but low for Asian. Those results should be replicated on larger groups of individuals.

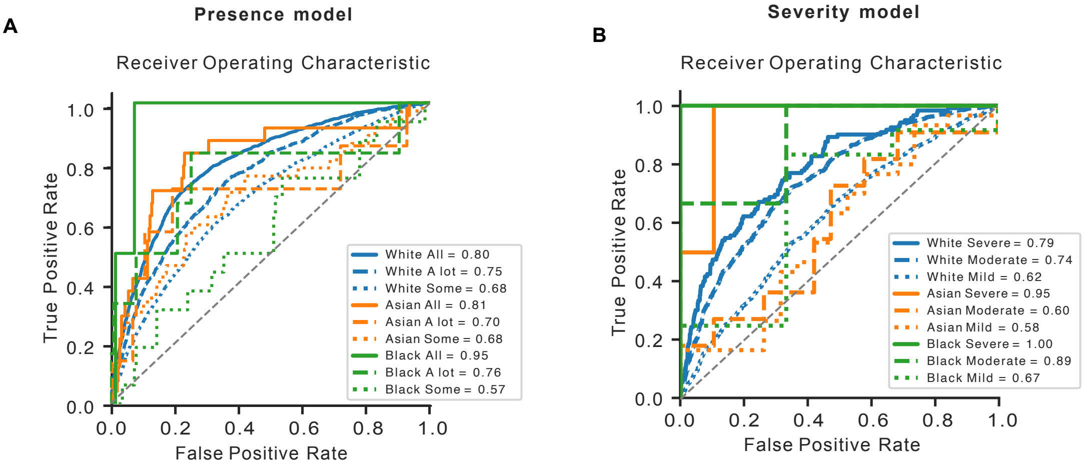

Figure S4: Odd ratios of tinnitus presence and tinnitus severity at follow-up, depending respectively on tinnitus presence and tinnitus severity at baseline

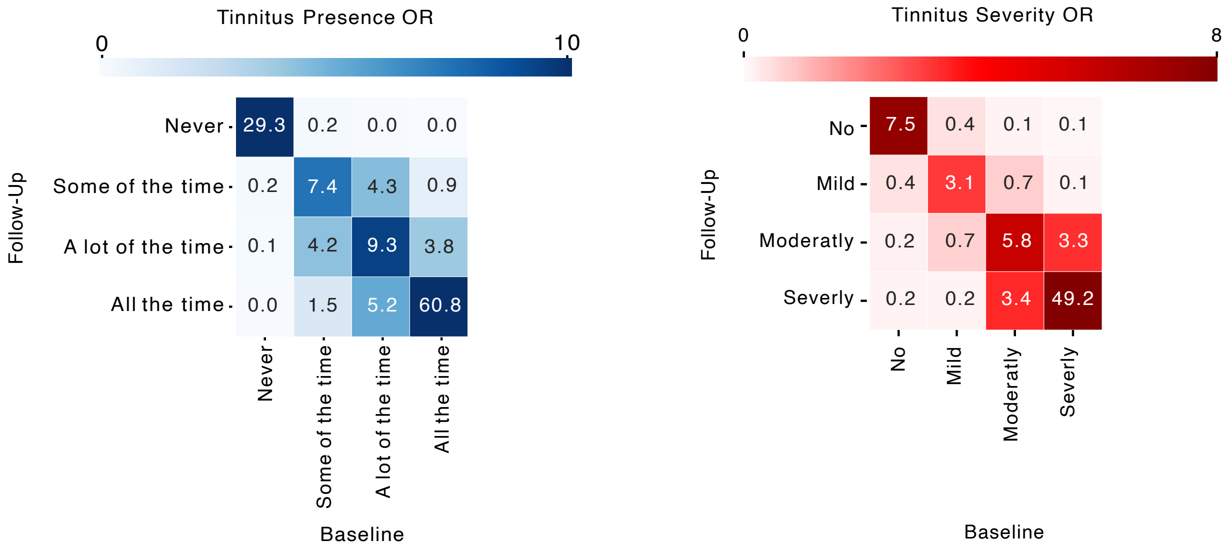

Table S1: Number of participants in longitudinal dataset. The table depicts the number of participants for each combination of tinnitus presence at the baseline visit, and tinnitus presence at the follow-up visit

		Presence at baseline			
		No	Some of the time	A lot of the time	Most or all the time
Presence at follow-up	No	19,483	1,005	115	100
	Some of the time	1,293	894	247	171
	A lot of the time	299	264	180	196
	Most or all the time	587	330	280	1,430

Table S2: Number of participants in longitudinal dataset. The table depicts the number of participants for each combination of tinnitus severity at the baseline visit, and tinnitus severity at the follow-up visit

		Severity at baseline			
		No	Mild	Moderate	Severe
Severity at follow-up	No	983	587	64	3
	Mild	530	1705	390	17
	Moderate	75	384	387	50
	Severe	9	20	57	58

Figure S5: Adjustment of the risk scores for the longitudinal evaluations.

We examined the prognostic value of the severity risk score by correcting for the severity at baseline. To this aim, we regressed out the squared values of the tinnitus presence level (0: No, 1: Some of the time, 2: A lot of the time, 3: All of the time) at baseline from presence the risk score calculated at baseline. Making the score orthogonal to the Tinnitus presence level at baseline allowed us to interpret interindividual deviations in this adjusted score as risk of recovery or worsening of tinnitus presence at the follow-up visit. Similar analysis was performed on the severity risk score with the levels 0: No, 1: Mild, 2: Moderate and 3: Severe). The process is depicted in the following figure.

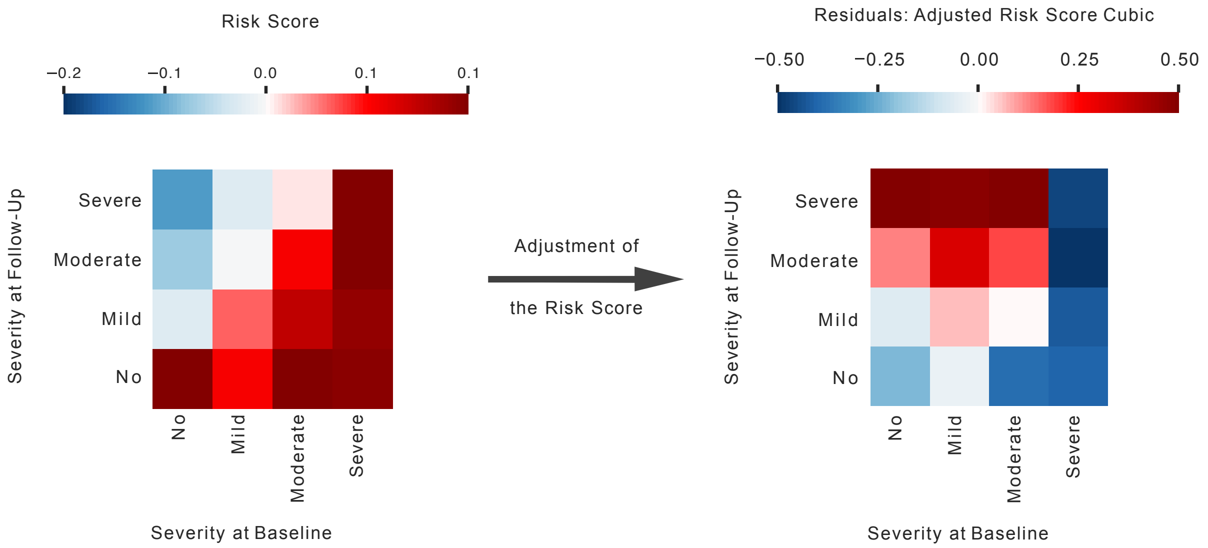

Figure S6. Examining the severity at follow-up depending on the reduced risk score at baseline

This figure depicts the odd ratios of experiencing a severe, moderate, mild or no distress associated with tinnitus at the follow-up visit, depending on the Reduced risk score at baseline. Based on those odd ratios, we can conclude that 0 and 1 risk scores are associated with a low risk of experiencing moderate or severe tinnitus over time. Scores 2 and 3 are associated with a moderate risk, score 4 and 5 are associated with a large risk and score 6 with a very high risk of experiencing moderate or severe tinnitus distress over time.

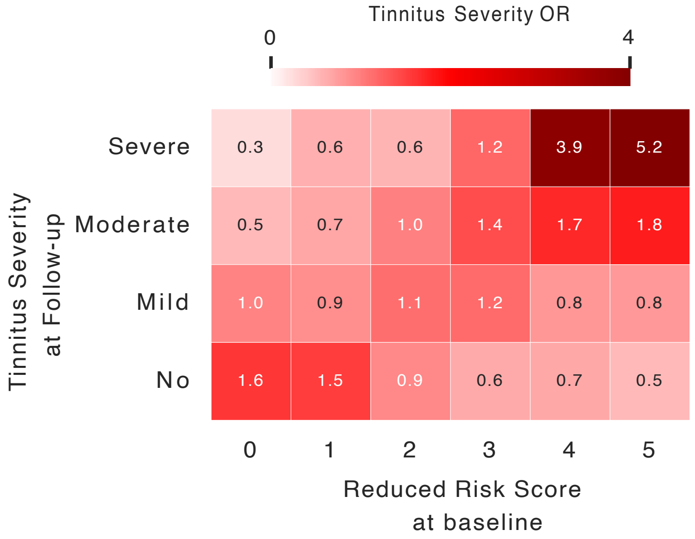

Figure S7: Flowchart describing the constitution of the presence model and the severity model datasets.

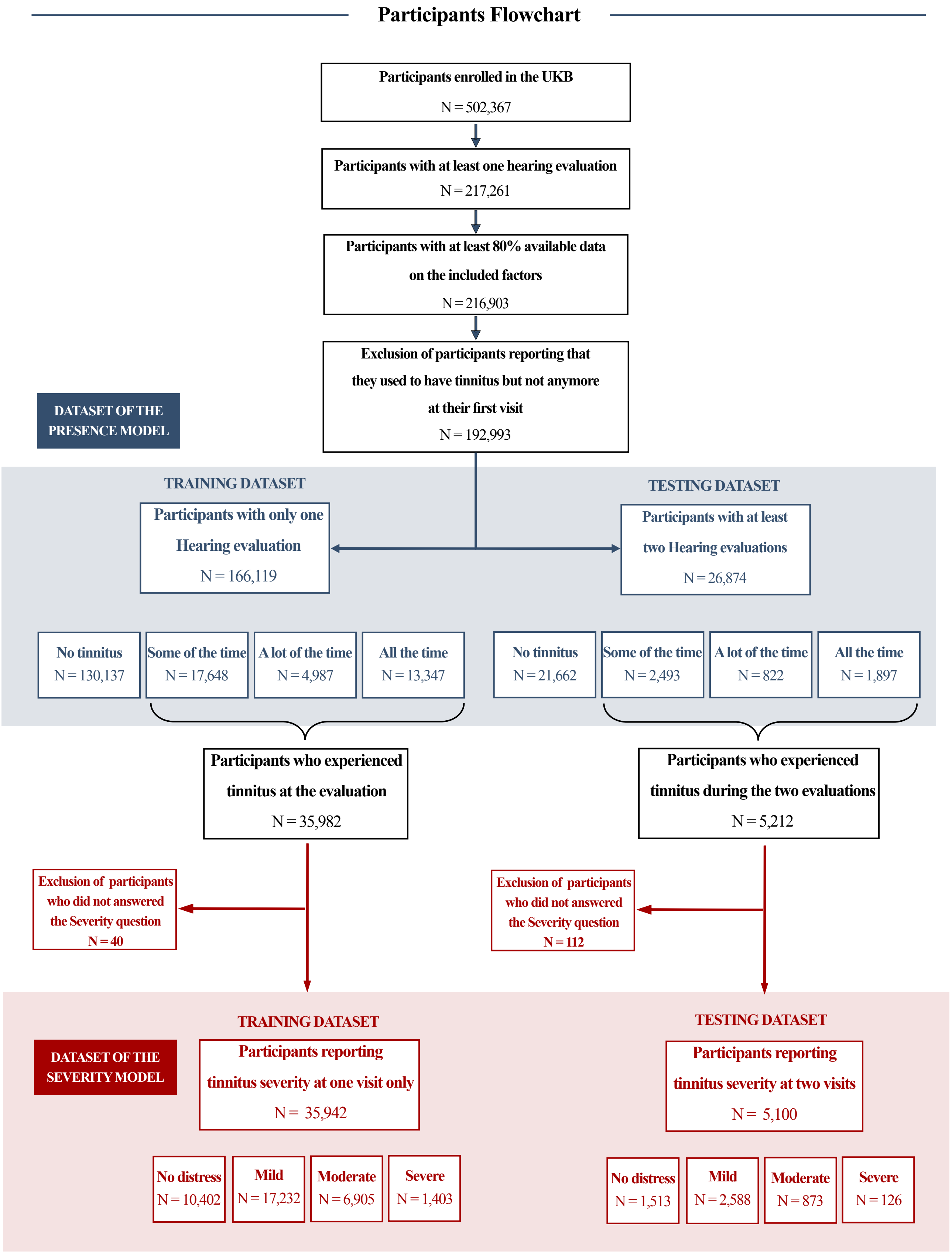

Tables S3: Constitution of the dataset of 217,261 participants out of the 502,367 participants of the UK Biobank.

The UK Biobank is constituted of one baseline visit (T0, n = 502,367 participants) and three follow-up visits for which a different subset of participants was included (T1: n = 20,343 participants, T2: n = 65,655 participants and T3: n = 5,357 participants). Hearing evaluation was not included in the dataset at the start of the data collection, thus only a sub-sample of participants at T0 were evaluated for their audition. To add as many participants as possible in our analysis, we added data of all participants who had at least one hearing evaluation in one of the visits. Table **A** describes the number of participants included in our analysis who had **only one** hearing evaluation. We described the visit of the UK Biobank at which they received this evaluation. If a participant had **at least two** hearing evaluations, we included the longitudinal evaluation in our dataset. Table **B** describes the number of participants who received at least two hearing evaluation, and at which UK Biobank visits they were performed. When participants had 3 or 4 hearing evaluations, we always kept the older evaluation as baseline, and the one following as a follow-up. For example, if a participant was evaluated at T0, T2 and T3, we included his evaluation at T0 as the baseline evaluation, and T2 as the follow-up evaluation.

Data at T0 were collected between April 2009 and October 2010. Data at T1 were collected between August 2012 and June 2013. Data at T2 were collected between April 2014 and march 2023. Finally, data at T3 were collected between June 2019 and February 2023

A. Number of participants with only one hearing evaluation

Visit	
T0	145,025
T1	8,631
T2	33,544
T3	47

B. Number of participants with at least two hearing evaluations

First visit (Baseline)	Second visit (Follow-up): T1	T2	T3
T0	4,625	18,568	20
T1		6,791	10
T2			0

Table S4: Variables extracted from the UK Biobank and their associated data field.

Type of measure	Details	UK Biobank Data Field
Tinnitus evaluation		
Tinnitus		4803
Tinnitus severity/nuisance		4814
Hearing		
Hearing difficulties self-reported		2247
Hearing difficulties self-reported with background noise		2257
Hearing test right ear		20021
Hearing test left ear		20019
Hearing aid		3393
Cochlear implants		4792
Noisy workplace		4825
Loud music exposure		4836
Mood		
Frequency of depressed mood in last 2 weeks		2050
Frequency of unenthusiasm/disinterest in last 2 weeks		2060
Frequency of tenseness/restlessness in last 2 weeks		2070
Frequency of tiredness/lethargy in last 2 weeks		2080
Seen doctor (GP) for nerves, anxiety, tension or depression		2090
Seen psychiatrist for nerves, anxiety, tension or depression		2100
Risk taking	Whether the participant describes themselves as someone who takes risks	2040
Neuroticism		
Neurotic behaviors	Mood swings	1920
	Miserableness	1930
	Irritability	1940
	Sensitivity/hurt feelings	1950
	Fed-up feelings	1960
	Nervous feelings	1970
	Worrier/anxious feelings	1980
	Tens/highly strung	1990
	Worry too long after embarrassment	2000
	Suffer from nerves	2010
	Loneliness	2020
	Guilty feeling	2030
Total neuroticism score	Derived from the 12 neurotic behaviors, as a sum score of the total "yes" answers to these questions	20127
Life stressors		
Serious illness, injury, bereavement in the last 2 years	Serious trauma to self<br>Serious trauma to close relative<br>Death of close relative<br>Death of spouse or partner<br>Martial separation/ divorce<br>Financial difficulties	6145
Life stressors whithin last 2 years	Derived from the life stressors 6145, as a sum score of the total "yes" answers to these questions	-
Sleep		
Sleep duration	Hours of sleep in every 24 hours	1160

Getting up in the morning	Difficulty getting up	1170
Sleeplessness/insomnia	Trouble falling asleep at night or waking up in the middle of the night	1200
Nap during the day		1190
Chronotype	Late chronotype (evening person)<br>Early chronotype (morning person)	1180
Daytime dozing (narcolepsy)	Likely to doze off or fall asleep during the daytime	1220
Physical activity		
Hand grip strength	Average between left- and right-hand grip strength (units of measurement: Kg).	46 & 47
IPAQ activity group	Low<br>Moderate<br>High	22032
MET minutes per week for walking	Units of measurement: minutes/week	22037
MET minutes per week for moderate activity	Units of measurement: minutes/week	22038
Above moderate/vigorous recommendation	Indicates if the participant met the 2017 UK Physical activity guidelines of 150 minutes of moderate activity per week or 75 minutes of vigorous activity	22035
Substance Use		
Smoking status	Previous smoker	20116
Current tobacco smoking	Daily smoker<br>Occasional smoker	1239
Past tobacco smoking	How often the participant smoked tobacco in the past	1249
Ever smoked	Derived from current and past tobacco smoking status (data fields 1239 & 1249)	20160
Smokers in household	If anyone smokes in the participant's household	1259
Hours of exposure to tobacco at home	Hours per week	1269
Alcohol drinker status	Never drank<br>Previous drinker	20117
Alcohol intake frequency		1558
Alcohol intake vs. 10 years previously	Indicates the change in the participant's alcohol use compared to 10 years ago	1628
Anthropometric		
Body mass index (BMI)	Constructed from height and weight measured during the in-person assessment visit	21001
Weight change compared with one year ago	Gained weight<br>Lost weight	2306
Weight	Unit of measurement: kg	21002
Systolic blood pressure	Units of measurement: mmHg	4080
Diastolic blood pressure	Units of measurement: mmHg	4079
Pulse rate	Pulse rate measured during the automated blood pressure readings (units of measurement: bpm).	102
Fractured/broken bones in last 5 years		2463
Occupational		
Job involves heavy manual or physical work		816
Job involves walking or standing		806
Current employment status	Looking after home or family<br>Paid employment/self-employed<br>Retired<br>Unemployed<br>Unable to work or disable	6142
Education (qualifications)	College or university<br>Advanced level	6138

	Ordinary level<br>Certificate secondary education<br>Practical career diploma<br>Other professional qualifications	
Demographics		
Ethic background	White<br>Asian<br>Black<br>Mixed<br>Other	21000
Sex	Female / Male	31
Age	Age in years	21003
Socioeconomics		
Number in household	Number of people living together in participant's household including themselves	709
Relationship with people living in household	Living with partner<br>Living with children<br>Living with siblings<br>Living with parents<br>Living with grandparents<br>Living with grandchildren<br>Living with related relatives<br>Living with unrelated relatives	6141
Frequency of friends and family visits		1031
Able to confide	How often the participant has been able to confide to someone close to them	2110
Number of vehicles in household		728
Average total household income		738

Figure S8: Comparison of two data imputation techniques: the median imputation and the Bayesian imputation method. We compared the accuracy of the presence (A) and severity (B) models depending on the data imputation methods, as well as the weights attributed to the features. We observe that the data imputation method did not significantly modify the models' accuracy, nor the weights attributed to each feature.

Data imputation
Comparison between different methods

A

Presence Model

ROC-AUC	Some of the time	A lot of the time	Most or all the time
Median Imputation	.68	.75	.80
Bayesian Imputation	.68	.75	.80

Model weights, in function of the data imputation method

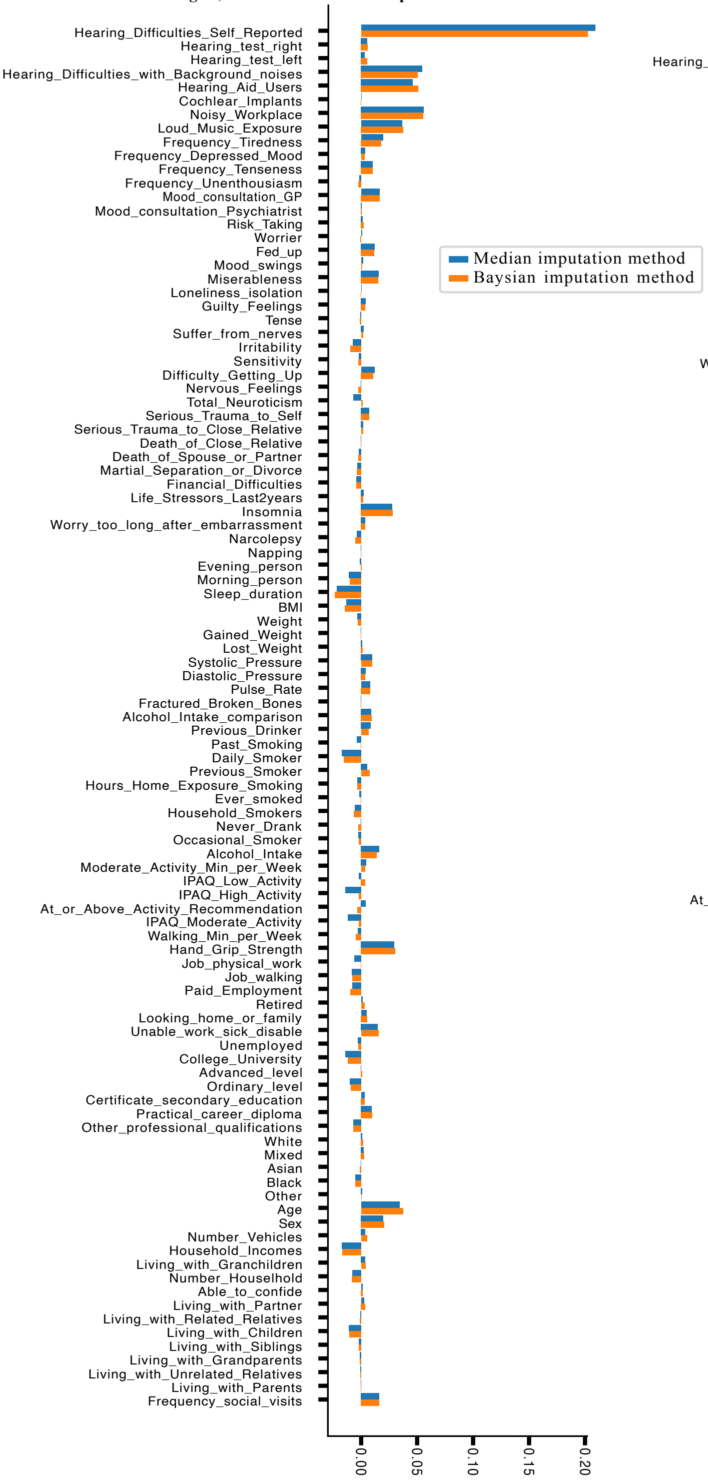

B

Severity Model

ROC-AUC	Mild	Moderate	Severe
Median Imputation	.62	.74	.79
Bayesian Imputation	.61	.74	.79

Model weights, in function of the data imputation method

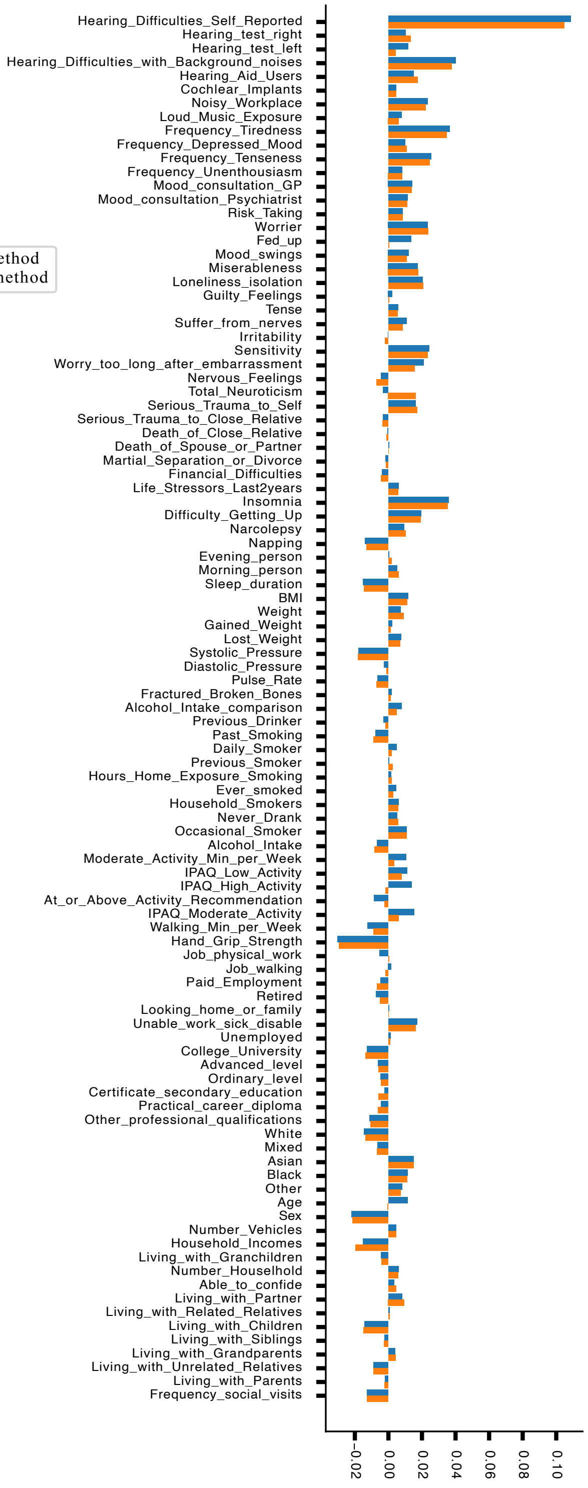

Supplementary Note S1. Developing the predictive models predicting tinnitus presence and severity

The nonlinear iterative partial least square (NIPALS) method was trained within the discovery dataset to identify latent scores (**E** and **Z**) and loadings (**P** and **H**) that maximize the covariance between matrix of standardized psychosocial features (X_i) (size (147385, 101) for tinnitus presence, and (43983, 101) for tinnitus severity) and a vector of self-reported tinnitus presence or severity (**Y**) (size (147385, 1) for tinnitus presence, and (43983, 1) for tinnitus severity):

1. Compute singular vectors **μ**, **ν** (weights) of covariance matrix $C=X^T\mathbf{Y}$
2. Obtain the latent scores **E** and **Z** by projecting X and **Y** onto singular vectors **μ** and **ν**
3. Compute loadings **P** and **H** by iteratively regressing X onto **E** (power iteration)
4. 4. Deflate X and **Y** using $X + 1 = X - \mathbf{EP}^T$ and $\mathbf{Y} + 1 = Y - \mathbf{ZH}^T$, respectively
5. 5. Fit training (discovery) data X using the projection matrix **P** to obtain latent space x̄ so that $\bar{x} = X\mathbf{P}$
6. Use the latent space to predict left-out data $\mathbf{Y_V}$ (size (20853,1) for tinnitus presence, (4291,1) for tinnitus severity) using the coefficient matrix $\beta \in R^{d \times t}$ β such that $\mathbf{Y_V} = \mathbf{X_V}\beta$, where $X_\mathbf{V}$ denotes the matrix of psychosocial features in the validation set ($X_\mathbf{V}$ size (20853,101) for tinnitus presence, (4291,101) for tinnitus severity)

Further information on the implementation can be found at https://scikit-learn.org/stable/modules/cross_decomposition. html#cross-decomposition.

Supplementary Note S2. Reduced risk score replication: TRI Database

The reduced severity risk score was derived using six binarized items:

1. **Do you think you have a hearing problem?** 0: No / 1: Yes
2. **Do you have a problem tolerating sounds because they often seem much too loud? That is, do you often find tool loud or hurtful sounds which other people around you find quite comfortable?** 0: No, 0: Rarely, 0: Sometimes / 1: Usually, 1: Always
3. **How much of the time have you had trouble sleeping at night?** 0: At no time, 0: Some of the time, 0: Slightly less than half the time, 0: Slightly more than half the time/ 1: Most of the time, 1: All the time
4. **How much of the time have you felt lacking in energy and strength?** 0: At no time, 0: Some of the time, 0: Slightly less than half the time, 0: Slightly more than half the time/ 1: Most of the time, 1: All the time
5. **How much of the time have you felt low in spirits or sad?** 0: At no time, 0: Some of the time, 0: Slightly less than half the time, 0: Slightly more than half the time/ 1: Most of the time, 1: All the time
6. **How much of the time have you had a bad conscience or feelings of guilt?** 0: At no time, 0: Some of the time, 0: Slightly less than half the time, 0: Slightly more than half the time/ 1: Most of the time, 1: All the time

The TRI database did not contain information on speech-in-noise hearing difficulties, which could be a proper equivalent of the original item "Do you find it difficult to follow a conversation if there is background noise (such as TV, radio, children playing)?". We replaced it by an evaluation of hyperacusis, i.e. hypersensitivity to sounds, a symptom often linked to tinnitus and hearing deficits as speech-in-noise hearing deficits. Questions 5) and 6) of the questionnaire POST were replaced here by other items evaluating mental health.
