# Supplementary material for: Tinnitus risk factors and its evolution over time
Source: Nat Commun. 2025 May 7;16:4244. doi: 10.1038/s41467-025-59445-3 (PMC12059016; doi:10.1038/s41467-025-59445-3)
Supplement: Supplementary file 2 — Reporting Summary [file 41467_2025_59445_MOESM2_ESM.pdf]

nature portfolio

Corresponding author(s): Lise Hobeika and Etienne Vachon-Presseau

Last updated by author(s): Sep 3, 2024

Reporting Summary

Nature Portfolio wishes to improve the reproducibility of the work that we publish. This form provides structure for consistency and transparency in reporting. For further information on Nature Portfolio policies, see our Editorial Policies and the Editorial Policy Checklist.

Statistics

For all statistical analyses, confirm that the following items are present in the figure legend, table legend, main text, or Methods section.

n/a	Confirmed	
☐	☒	The exact sample size (*n*) for each experimental group/condition, given as a discrete number and unit of measurement
☐	☒	A statement on whether measurements were taken from distinct samples or whether the same sample was measured repeatedly
☐	☒	The statistical test(s) used AND whether they are one- or two-sided<br>*Only common tests should be described solely by name; describe more complex techniques in the Methods section.*
☐	☒	A description of all covariates tested
☐	☒	A description of any assumptions or corrections, such as tests of normality and adjustment for multiple comparisons
☐	☒	A full description of the statistical parameters including central tendency (e.g. means) or other basic estimates (e.g. regression coefficient) AND variation (e.g. standard deviation) or associated estimates of uncertainty (e.g. confidence intervals)
☒	☐	For null hypothesis testing, the test statistic (e.g. *F*, *t*, *r*) with confidence intervals, effect sizes, degrees of freedom and *P* value noted<br>*Give P values as exact values whenever suitable.*
☒	☐	For Bayesian analysis, information on the choice of priors and Markov chain Monte Carlo settings
☒	☐	For hierarchical and complex designs, identification of the appropriate level for tests and full reporting of outcomes
☐	☒	Estimates of effect sizes (e.g. Cohen's *d*, Pearson's *r*), indicating how they were calculated

Our web collection on statistics for biologists contains articles on many of the points above.

Software and code

Policy information about availability of computer code

Data collection	No software was used for data collection in this study. Data was sourced from two publicly available datasets: the UK Biobank and the Tinnitus Research Initiative (TRI) database
Data analysis	Analyses were performed using Python v.3.11.5 with Spyder 5.4.3, including Numpy (v.1.24.3), Pandas (v.2.0.3), Sklearn (v.1.3.0), Seaborn (v.0.12.2), Matplotlib (v.3.7.2), Pingouin (v.0.5.3) and Nltools (v.0.5.0).

For manuscripts utilizing custom algorithms or software that are central to the research but not yet described in published literature, software must be made available to editors and reviewers. We strongly encourage code deposition in a community repository (e.g. GitHub). See the Nature Portfolio guidelines for submitting code & software for further information.

Data

Policy information about availability of data

All manuscripts must include a data availability statement. This statement should provide the following information, where applicable:

- Accession codes, unique identifiers, or web links for publicly available datasets
- A description of any restrictions on data availability
- For clinical datasets or third party data, please ensure that the statement adheres to our policy

The dataset from UK Biobank analyzed in the study is available via application to the Access Management System at https://www.ukbiobank.ac.uk. The TRI dataset is accessible upon requests, see https://tinnitusresearch.net/index.php/for-researchers/tinnitus-database.

Research involving human participants, their data, or biological material

Policy information about studies with human participants or human data. See also policy information about sex, gender (identity/presentation), and sexual orientation and race, ethnicity and racism.

Reporting on sex and gender	The sex of participants, as reported in the UK Biobank, was included in the models as potential risk factors. It is important to note that no information regarding gender identity was collected.
Reporting on race, ethnicity, or other socially relevant groupings	The study cohort consists of the general population from the United Kingdom with a predominance of white ethnicity (94-96%). We validated the risk scores independently for groups of participants with Asian ethnicities, and Black ethnicities on the most severe diagnosis even if the sample sizes were small (ROC-AUC > .71 for all the time vs no tinnitus; and ROC-AUC > .71 for severe tinnitus vs no distress).
Population characteristics	The study cohort consists of the general population from the United Kingdom, aged 40-70 years at the baseline visit, with 51-55% of participants being female and predominantly of white ethnicity (94-96%). Participants provided electronic health records that detail current and past clinical diagnoses or provided self-reported medical history. In all machine learning models, age and sex were controlled to reliably estimate the biological effects stemming from the disease process.
Recruitment	*Describe how participants were recruited. Outline any potential self-selection bias or other biases that may be present and how these are likely to impact results.*
Ethics oversight	The UKB was approved by the Research Ethics Committee (no. 11/NW/0382). Ethical approval for the collection of the TRI database was obtained from the Ethics Committee of the University of Regensburg (protocol number 08/046). All participants gave written, informed consent.

Note that full information on the approval of the study protocol must also be provided in the manuscript.

Field-specific reporting

Please select the one below that is the best fit for your research. If you are not sure, read the appropriate sections before making your selection.

☒ Life sciences ☐ Behavioural & social sciences ☐ Ecological, evolutionary & environmental sciences

For a reference copy of the document with all sections, see nature.com/documents/nr-reporting-summary-flat.pdf

Life sciences study design

All studies must disclose on these points even when the disclosure is negative.

Sample size	No sample-size calculation was done. A total of 147,133 participants of the UK Biobank were included in this study. The replication was done on 463 individuals of the TRI database
Data exclusions	On the UK Biobank, participants with more than 20% of missing features were excluded. No other exclusion criteria were applied to ensure that the study findings would be as generalizable as possible to the wider population. On the TRI dataset, only participants with longitudinal evaluation were included.
Replication	Models were derived and validated within the UK Biobank cohort using cross-validation to ensure robustness.
Randomization	Randomization was incorporated into the models through the cross-validation process, which involved shuffling the data before splitting participants into various training and testing folds
Blinding	Blinding was not necessary for this study, as the determination of feature importance was conducted algorithmically through the machine learning models.

Reporting for specific materials, systems and methods

We require information from authors about some types of materials, experimental systems and methods used in many studies. Here, indicate whether each material, system or method listed is relevant to your study. If you are not sure if a list item applies to your research, read the appropriate section before selecting a response.

Materials & experimental systems

n/a	Involved in the study
☒	☐ Antibodies
☒	☐ Eukaryotic cell lines
☒	☐ Palaeontology and archaeology
☒	☐ Animals and other organisms
☒	☐ Clinical data
☒	☐ Dual use research of concern
☒	☐ Plants

Methods

n/a	Involved in the study
☒	☐ ChIP-seq
☒	☐ Flow cytometry
☒	☐ MRI-based neuroimaging

Plants

Seed stocks

Report on the source of all seed stocks or other plant material used. If applicable, state the seed stock centre and catalogue number. If plant specimens were collected from the field, describe the collection location, date and sampling procedures.

Novel plant genotypes

Describe the methods by which all novel plant genotypes were produced. This includes those generated by transgenic approaches, gene editing, chemical/radiation-based mutagenesis and hybridization. For transgenic lines, describe the transformation method, the number of independent lines analyzed and the generation upon which experiments were performed. For gene-edited lines, describe the editor used, the endogenous sequence targeted for editing, the targeting guide RNA sequence (if applicable) and how the editor was applied.

Authentication

Describe any authentication procedures for each seed stock used or novel genotype generated. Describe any experiments used to assess the effect of a mutation and, where applicable, how potential secondary effects (e.g. second site T-DNA insertions, mosiacism, off-target gene editing) were examined.
